# Multi-omics reveals EGCG’s anti-calcification effects associated with gut microbiota and metabolite remodeling

**DOI:** 10.3389/fimmu.2025.1595527

**Published:** 2025-06-26

**Authors:** Yating Zhang, Zihan Tang, Junwen Zhu, Ruochi Zhao, Shuangshuang Wang

**Affiliations:** ^1^ Department of Cardiology, The First People’s Hospital of Wenling, Wenling Hospital of Wenzhou Medical University, Wenling, Zhejiang, China; ^2^ College of Bioscience and Biotechnology, Hunan Agricultural University, Changsha, China; ^3^ Key Laboratory of Precision Medicine for Atherosclerotic Diseases of Zhejiang Province, Affiliated First Hospital of Ningbo University, Ningbo, China

**Keywords:** epigallocatechin-3-gallate, natural products, vascular calcification, gut microbes, serum metabolites

## Abstract

**Introduction:**

Vascular calcification, a pathological process driven by heterotopic calcium-phosphate deposition, arises from vascular smooth muscle cells (VSMCs) osteochondrogenic transformation, epigenetic dysregulation, and metabolic reprogramming. Epigallocatechin-3-gallate (EGCG), a natural polyphenol, is associated with attenuated vascular calcification and remodeling of the gut microbiota-metabolite axis.

**Methods:**

Twenty-four 8-week-old Sprague-Dawley rats were randomized into four groups: control (CON), vitamin D3-induced calcification (VD), VD plus EGCG (VD+EGCG), and EGCG-only (EGCG). Vascular calcification was induced via vitamin D3 injection, followed by 11-week EGCG treatment. Calcification severity was quantified using alizarin red S staining, alkaline phosphatase (ALP) immunohistochemistry/immunofluorescence, and serum metabolomics, while colon microbiota and metabolites were profiled via 16S rRNA sequencing and LC-MS/MS.

**Results:**

EGCG significantly reduced calcification (*P<0.05 vs. VD), as evidenced by diminished alizarin red S staining and suppressed ALP activity. Gut microbiota analysis revealed EGCG-mediated restoration of alpha diversity and taxonomic shifts, including reversal of Spirochaetota, Desulfobacterota, and Actinobacteriota abundances at the phylum level (*P<0.05); marked changes in Clostridia_UCG_014, Desulfovibrionales, Christensenellales, Erysipelotrichales, Oscillospirales, and Spirochaetales at the order level (*P<0.05); and normalization of *Treponema, unclassified Treponema*, and *Lactobacillus johnsonii* at the genus/species level (*P<0.05). Serum metabolomics identified VD3-induced upregulation of phospholipid metabolites (phosphatidylserine [PS], phosphatidylcholine [PC], lysophosphatidylcholine [LysoPC]), which were counteracted by EGCG (*P<0.05). Concurrently, EGCG enhanced ubiquinone biosynthesis and terpenoid-quinone pathways.

**Discussion:**

These changes are mechanistically linked to suppressed VSMCs osteogenic differentiation and aortic degeneration. The findings establish EGCG as a dual microbiota-metabolite modulator with therapeutic potential for vascular calcification, offering a novel strategy to target gut-vascular crosstalk in cardiovascular disease.

## 1 Introduction

Vascular calcification (VC) is an abnormal process of bone-specific hydroxyapatite deposition, mainly in the form of calcium phosphate crystals, on the vessel wall. Vascular calcification is currently considered to be an active osteogenic process of blood vessel cells (mainly vascular endothelial cells), which can be classified into three phases: differentiation of osteoblasts, maturation of the matrix and mineralization of the matrix, similar to the formation of osteoblasts (1). Osteogenic differentiation characterized by Vascular smooth muscle cells (VSMCs) increases the expressed bone markers and simultaneously diminishes the expressed VSMCs markers (2–4). VSMCs are a multipotent type of cell in the vasculature that display a constrictive phenotype physiologically. Vascular smooth muscle cells are an essential part of the vascular wall, providing architectural support, modulating vascular tone, and reshaping the vasculature (5). VSMCs can differentiate into osteoblasts, which are thought to be the initiating stage of vascular calcification (6). VSMCs-originated osteoblasts are capable of secreting the enzyme alkaline phosphatase (ALP), and ALP is incorporated into vesicles to facilitate the liberation of free phosphates, setting the stage for mineralization of tissues and cells (7).

Although VC is a key contributor to cardiovascular disease mortality, there is no effective way to reverse the progression of VC. Therefore, recent therapeutic strategies have focused more on inhibiting or delaying the progression of VC (8). There are not many medications available for the treatment of vascular calcification, so there is an increasing interest in the dietary aspects of preventing or slowing down vascular calcification.

The definition of a prebiotic is “a non-digestible substrate that can be selectively utilized by host microorganisms to confer a health benefit” (9). Epigallocatechin-3-gallate (EGCG) is a monomeric polyphenol compound derived from green tea and is the main component responsible for the pharmacological effects of green tea. As a “prebiotic”, EGCG has been shown in numerous studies to play an active role in the treatment of cardiovascular diseases by attenuating acute myocardial infarction (10), ameliorating cardiac hypertrophy (11) and heart failure (12), and exerting anti-atherogenic effects (13). Rebecca L Noad et al. conducted the Polyphenol Intervention Trial (PPhIT) in 92 patients with cardiovascular disease, where increasing dietary polyphenols through consumption of F&V, berries and dark chocolate significantly improved established cardiovascular risk markers in hypertensive participants, with a significantly greater increase in epicatechin in the high-polyphenol group (p=0.008), suggesting a potential therapeutic role for EGCG in cardiovascular disease (14). Furthermore, the beneficial effects of prebiotics need to be caused in part by microbial changes. EGCG cannot be metabolized and absorbed without the involvement of the intestinal flora, and the metabolites of EGCG can have even more favorable effects on the intestinal flora and on the human body (15). The healthy intestinal microbiota contributes to the maintaining of intact intestinal barrier features, which are closely related to the health status of the host (16). EGCG as a polyphenol can remodel the gut microbiota to enhance host-microbe interactions (17). The composition of the intestinal flora affects the bioavailability of polyphenols and their metabolites (18). Modulation of intestinal flora increases sugar metabolism, improves intestinal barrier function and energy expenditure, and reduces inflammation, insulin resistance, obesity, weight gain, and dyslipidemia.

EGCG has been overlooked by most researchers due to its bioavailability, and there have been no FDA approved drugs to treat or invert vascular calcification. The aim of this study was the therapeutic potential of EGCG in vascular calcification disease. Vascular calcification was induced in rats by subcutaneous injection of vitamin D3, and in the treatment group, epigallocatechin-3-gallate (EGCG) was administered orally. 84 days later, the extent of vascular calcification, ALP activity, and changes in the gut microbiota and metabolic profiles were analyzed using high-throughput sequencing of intestinal contents and serum metabolomics, and the therapeutic efficacy of EGCG on vascular calcification disease was determined.

## 2 Methods

### 2.1 Experimental design

All animal procedures strictly followed the Laboratory Animal Care and Utilization Guidelines established by Hunan Agricultural University, with formal approval (2024134) granted by the Animal Protection and Wellness Committee. SPF SD rats (8 weeks old) were housed under controlled SPF conditions, and the experimental animals were obtained from Hunan Slake Jinda Laboratory Animal Co. Vitamin D3 was purchased from Haotian Biomaterials (Zhuang City, Zhejiang Province, China), and EGCG was purchased from Nanjing Daosifu Biotechnology Co(Nanjin, China).

**Figure 1:**
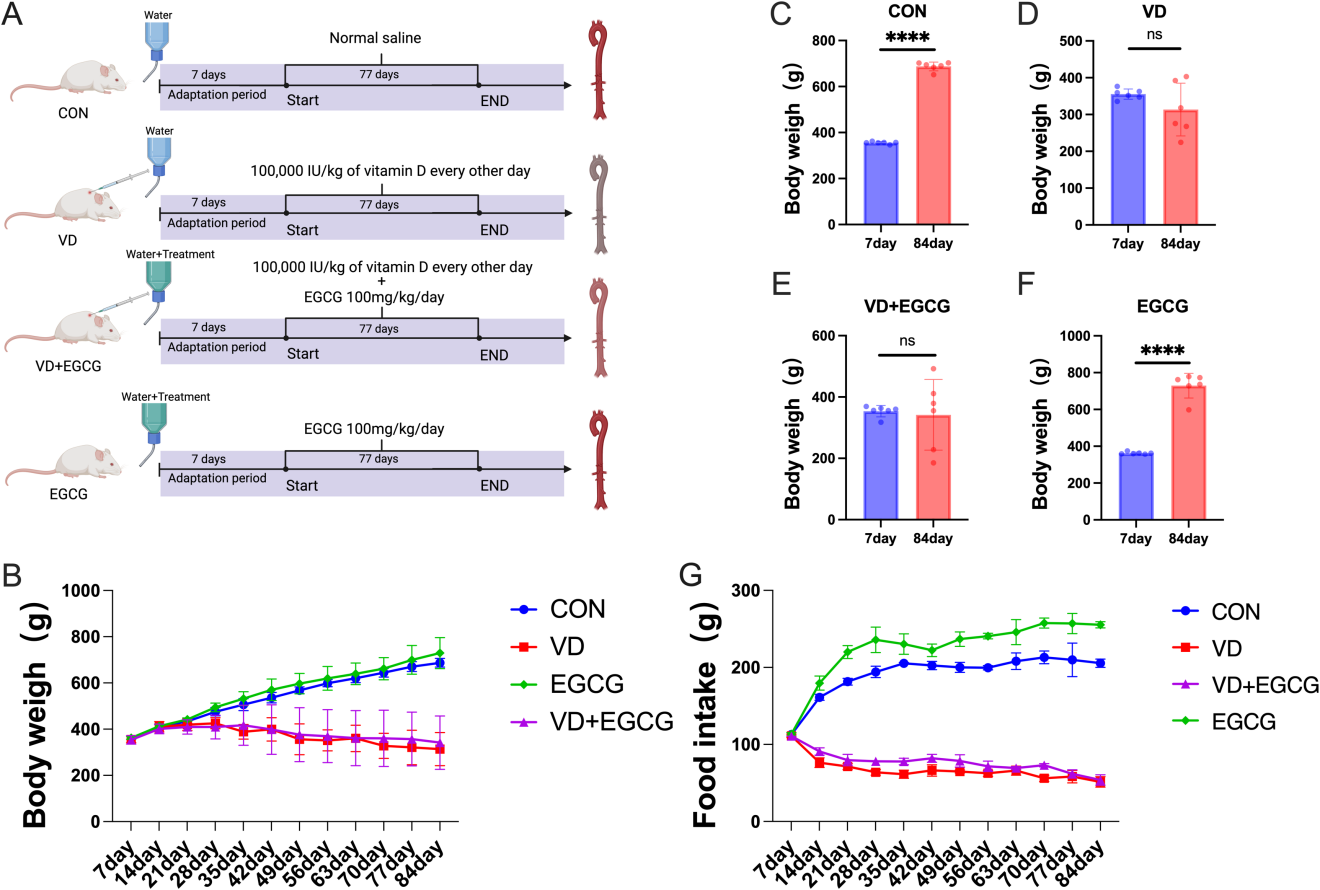
Overview of the experimental design. **(A)** Assignment of laboratory groups and workflow: 24 rats were assigned to four different groups (CON, VD, VD+EGCG and EGCG) for 84 days. **(B)** Body weight fluctuation of rats in the four groups: CON group (n = 6), VD group (n = 6), VD+EGCG group (n = 6), and EGCG group (n = 6). **(C)** Comparison of body weights of rats in CON group on day 7 and day 84. **(D)** Comparison of body weight on day 7 and day 84 in rats of VD group. **(E)** Comparison of body weight on day 7 and day 84 in rats of VD+EGCG group. **(F)** Comparison of body weight between day 7 and day 84 in rats of VEGCG group. **(G)** Food intake of each group. Results are presented as mean ± SE, determined by one-way ANOVA and Tukey’s HSD and LSD-t test. ****P < 0.001; ns > 0.05.

All the rats used in this experiment were maintained in sterile environments with ad libitum access to nutrition and hydration (ambient conditions: 24 ± 2°C, 50 ± 5% humidity; photoperiod: 12h light/dark cycle). Following a 7-day acclimation period, subjects underwent stratified randomization into four experimental cohorts: (1) CON (control, basic diet, n=6), (2) VD (vitamin D3-induced vascular calcification, n=6), (3) VD+EGCG (vitamin D3 with epigallocatechin-3-gallate intervention, n=6), and (4) EGCG (basic diet plus epigallocatechin-3-gallate, n=6). Commencing on day 8, VD and VD+EGCG groups received alternate-day subcutaneous administrations of vitamin D3 (0.02 ml, 100,000 IU/kg) over 11 consecutive weeks. Daily oral supplementation with 100 mg/kg epigallocatechin-3-gallate was implemented in both VD+EGCG and EGCG groups via drinking water. Notable physiological responses to vitamin D3 administration emerged within the first week. Longitudinal monitoring included weekly body weight measurements, tri-daily food/water consumption records, and dose adjustments based on weight fluctuations. Prior to terminal procedures at week 11, animals underwent 12-hour fasting while maintaining free water access. Final sample collection was conducted through humane euthanasia to complete the experimental protocol (**Figure 1A**).

### 2.2 Sample collection

Sampling consisted of intravenous injection of chloral hydrate at a concentration of 10% to anesthetize the rats, and then blood samples were collected by cardiac puncture. Centrifugation at 4°C and 1500r for 5 minutes separates the serum, which is then storaged at -80°C in readiness for analytical metabolomics. The cervix of the rat is then subluxated and killed. Collect 1–2 cm of colon matter and store it in labeled EP tubes. At -80°C they are promptly transferred to a refrigerator for 16S rRNA analysis of intestinal microorganisms. Aortic vessels from rats were autopsied and separated from the body, fixed with paraformaldehyde after removal of adherent fat and flushing of the thrombus, and then analyzed by alizarin red S staining.

### 2.3 Alizarin red S staining and paraffin section

After removing the rat aortas, the aortas were rinsed 3 times with PBS and fixed with 4% formaldehyde for 10 minutes. Then the rat aortas were washed 3 times with PBS, stained with Alizarin Red S Stain (0.1%, pH 8.3, Solarbio, Beijing, China) for 5 minutes, observed and photographed. After photographing, the tissue was immersed in 80%, 90%, 95%, and 100% various concentrations of ethanol for 2 hours. The paraffin wax was immersed into the tissue to replace the hyaluronan contained in the tissue, and the wax-immersed tissue was embedded in melted solid paraffin. Slicing; spreading; patching and baking: after the slices were fully spread out on the thermostatic water surface, the wax slices were fished to the middle section of the slide to pour off the remaining water on the slide and were placed into the thermostat at 60-65°C or the oven of the slicing bleaching and baking temperature controller to bake the slices for 15∼30 minutes, to take off paraffin wax from the interstitial space of the melted tissues. Afterwards, they were examined with a microscope (DM3000, Leica).

### 2.4 Immunohistochemistry and immunofluorescence

60°C × 20min→ xylene 2 × 10minutes; 100%absolute ethanol: 2 × 5minutes; 95% ethanol 2minutes; 80% ethanol 2minutes; 70% ethanol 2minutes; double-distilled water: 5minutes; PBS washed 3 times × 3minutes. Sections were infiltrated with a closed permeabilizing solution for 30minutes (RT to avoid light). Dispensing was done by heating 40ml PBS with 120ul TritonX-100 for a few minutes and then adding 400ul 30% H2O2 before use; the PBS solution was washed 3 times × 3min.The slices were placed in 0.01M sodium citrate buffer solution (pH=6.0) and then heated in a microwave oven on high for 4min until boiling, then heated for about 6min × 4 times, each time with intervals of replenishment of the liquid to prevent dry slices. PBS solution was washed 3 times × 3min;12 the sections were removed, the surrounding moisture was blotted dry with filter paper, a circle was drawn around the tissue with a histochemical pen, and a drop of 5% goat serum was added to the tissue within the circle (after agreeing with the source of the secondary antibody) flipped into the cassette at room temperature for 10-30min; the goat serum on the sections was shaken off, and the residual serum around the tissues was wiped dry with filter paper, and the diluted primary antibody was added directly (1: 250, 500, 1000), then put the sections into the wet box at room temperature for 1 hour, then 4 overnight, take the sections out from the refrigerator needs to be rewarmed at 37°C for 45min. Pour off the primary antibody and wash the sections with PBS for 5min×5 times; absorb the water around the circle with filter paper and put the sections into the oven at 37°C for 30min after adding the diluted secondary antibody (recycling); wash the sections with PBS for 5 times×5 min. add the SP after Put into the 37 degrees oven for 30min; wash with PBS 5 times × 5min add DAB (fast drop), observe the staining, pour off the staining solution, the time of color development was controlled at about 3~10min, the time of color control was controlled by microscopic observation. After 3 times×3min with PBS, wash with double-distilled water for 5min; add a large drop of hematoxylin staining solution, cytosolic proteins stained for a few seconds, cytoplasmic or cytosolic membrane proteins stained for 20s and then rinsed with tap water, double-distilled water washed for 5min, and then return to the blue with PBS for 5min.Dehydration: 50% ethanol 1-2min, 70% ethanol 1-2min, 95% ethanol 1-2min, 95% ethanol 1-2min Transparency: 1×xylene 1-2min→2×xylene 1-2min. sealing: neutral gum. Photographs were taken under a microscope (DM3000, Leica).

### 2.5 16S ribosomal RNA amplicon sequencing

Nucleic acids were detected by adding 1X dsDNA HS Working Solution (Next Sense Biotechnology Co., Ltd., Shanghai, China) to a Gene Compang Limited (Hong Kong, China) ELISA (synergy HTX) and amplified according to the concentration and amplification zone on a Bioline 1000 automated (Refudi Biomedical Co., Ltd., Shanghai, China). The amplified PCR products were analyzed by electrophoresis using 1.8% agarose (Beijing BMFX Technology Co., Ltd., Beijing, China) for fragmentation and integrity.

### 2.6 Liquid chromatography-mass spectrometry analysis

For serum metabolite analysis, 100 μL aliquots stored at -80°C underwent thawing and were subsequently homogenized with 300 μL methanol (Merck, Darmstadt, Germany) containing 10 μL DL-o-chlorophenylalanine (Sigma, St. Louis, MO) as internal standard. Following thorough vortexing, the mixture was subjected to 10-minute centrifugation at 12,000 rpm (4°C) using a refrigerated centrifuge. The clarified supernatant was then aseptically transferred to LC-MS vials using precision pipetting techniques. Chromatographic separation was achieved through Waters (Dublin, Ireland) LC-MS systems featuring an Ultra Gold C18 column (3 μm, 100 × 4.6 mm). Instrumental parameters and data processing workflows aligned with previously validated protocols (19). Time alignment and peak height correction by QC samples using Profinder (v 10.0) software is a classical metabolomics normalization strategy.

### 2.7 Statistical analysis

Statistical validation commenced with Levene’s test for homogeneity assessment, whereafter intergroup comparisons were determined by one-way ANOVA and Tukey’s HSD and LSD-t test. Significance and data analysis were performed using IBM SPSS Statistic 27.0 (SPSS, Inc., Chicago, USA). Samples were stained, and fluorescence was quantified using ImageJ (Mac OS X 10.8). Cohen’s d effect sizes were computed using JASP (Version 0.14.3) to quantify intergroup effect sizes. These analytical workflows were implemented using GraphPad Prism 10 (v10.0), which also facilitated microbiota-metabolite correlation studies. Across all inferential tests, statistical significance was defined by a two-tailed threshold of *P<0.05.

## 3 Results

### 3.1 Effects of epigallocatechin-3-gallate on vitamin D3-induced vascular calcification

Before the fourth week of vitamin D3 injection, the body weights of all groups were on an increasing trend, but after the fifth week of vitamin D3 injection, there was a decrease in both the VD group and the VD+EGCG group, and the body weights of the VD group and the VD+EGCG group became lighter with the longer duration of vitamin D3 injection (**Figure 1B**). Dietary intake differed significantly between the CON and VD groups. Notably, while EGCG intervention in the VD+EGCG group failed to ameliorate body weight loss compared to the VD group, this intergroup disparity was primarily attributable to differences in food intake (**Figure 1G**). No significant divergence in dietary consumption was observed between the VD and VD+EGCG groups. However, the EGCG group exhibited elevated food intake relative to the CON group. The lack of body weight recovery in VD+EGCG rats despite EGCG administration may reflect insufficient caloric compensation through dietary intake, though the underlying mechanisms remain unexplored.

**Figure 2:**
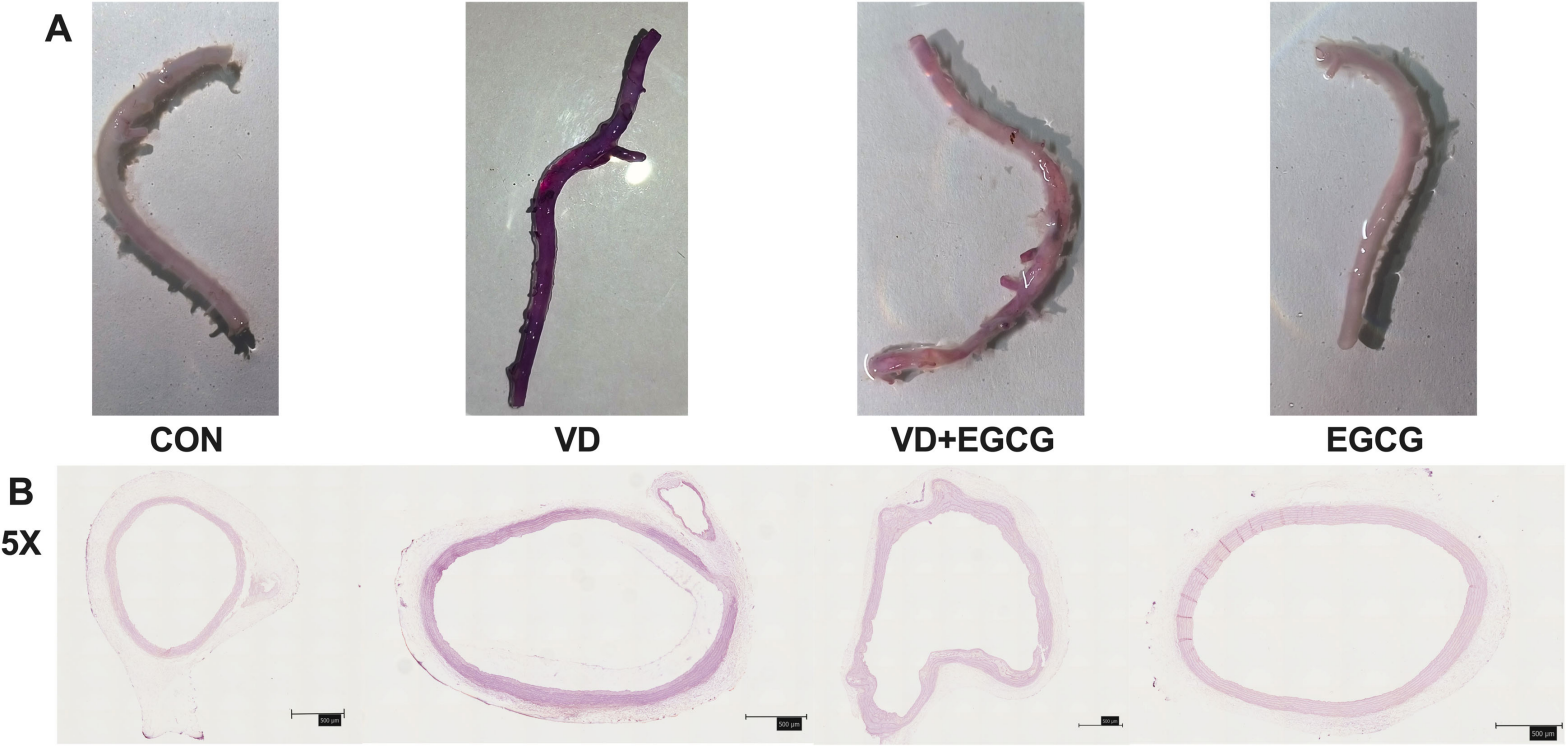
Rat aortic vascular staining map. **(A)** Alizarin red S-stained aortic angiograms of four groups of rats in CON, VD, VD+EGCG, and EGCG groups, Alizarin red S staining intensity exhibited a positive linear correlation with calcium deposition levels, where heightened purple-red chromogenic signals directly quantified pathological mineralization severity. **(B)** Calcification of the ascending aortic vasculature observed according to alizarin red dyeing of tissue sections (magnification 5×, 500μm).

Whole rat aortic vessels were stained with alizarin red S to assess calcification (**Figure 2A**). Qualitatively, the CON and EGCG groups exhibited minimal staining, closely resembling the native tissue coloration. In contrast, the VD group showed pronounced dark staining, distinct from other groups, while the VD+EGCG group exhibited intermediate staining intensity, reduced compared to the VD group (**Figure 2A**).

Quantitative analysis (**Table 1**) of alizarin red-stained sections included total fluorescence area (Area), mean intensity (Mean), minimum intensity (Min), maximum intensity (Max), and integrated density (IntDen). The VD group demonstrated significantly higher IntDen values compared to the CON and EGCG groups, whereas the VD+EGCG group showed reduced IntDen relative to the VD group (**Table 1**). Despite the VD group’s lower Mean intensity compared to other groups, as broader mineralization regions diluted the average intensity.

**Table 1:** Quantitative analysis of mineralization (alizarin red staining), immunofluorescence, and immunohistochemistry parameters in aortic tissue sections.

Targets	Groups	Area	Mean	Min	Max	IntDen
Alizarin Red S Slices	CON	53822	213.339	42	220	11482322
	VD	227106	202.98	69	217	46097961
	VD+EGCG	148839	218.047	214	221	32453891
	EGCG	114747	216.589	53	223	24852888
IF	CON(Blue)	19845120	2.805	0	255	55656499
	CON(Red)	19845120	2.922	0	255	57990028
	VD(Blue)	19845120	5.791	0	255	114930795
	VD(Red)	19845120	33.925	0	255	673238151
	VD+EGCG(Blue)	19845120	8.139	0	255	161520840
	VD+EGCG(Red)	19845120	9.904	0	255	196537055
	EGCG(Blue)	19845120	3.925	0	255	77887791
	EGCG(Red)	19845120	4.617	0	255	91615092
IHC	CON	228464	0.227	0.128	1.929	51956.433
	VD	380454	0.341	0.166	2.708	129594.882
	VD+EGCG	288349	0.196	0.114	0.944	56429.558
	EGCG	190681	0.227	0.128	0.901	43348.819

Fluorescence intensity, chromogen deposition, and calcium deposition were measured to assess alkaline phosphatase (ALP) activity and vascular calcification.

**Figure 3:**
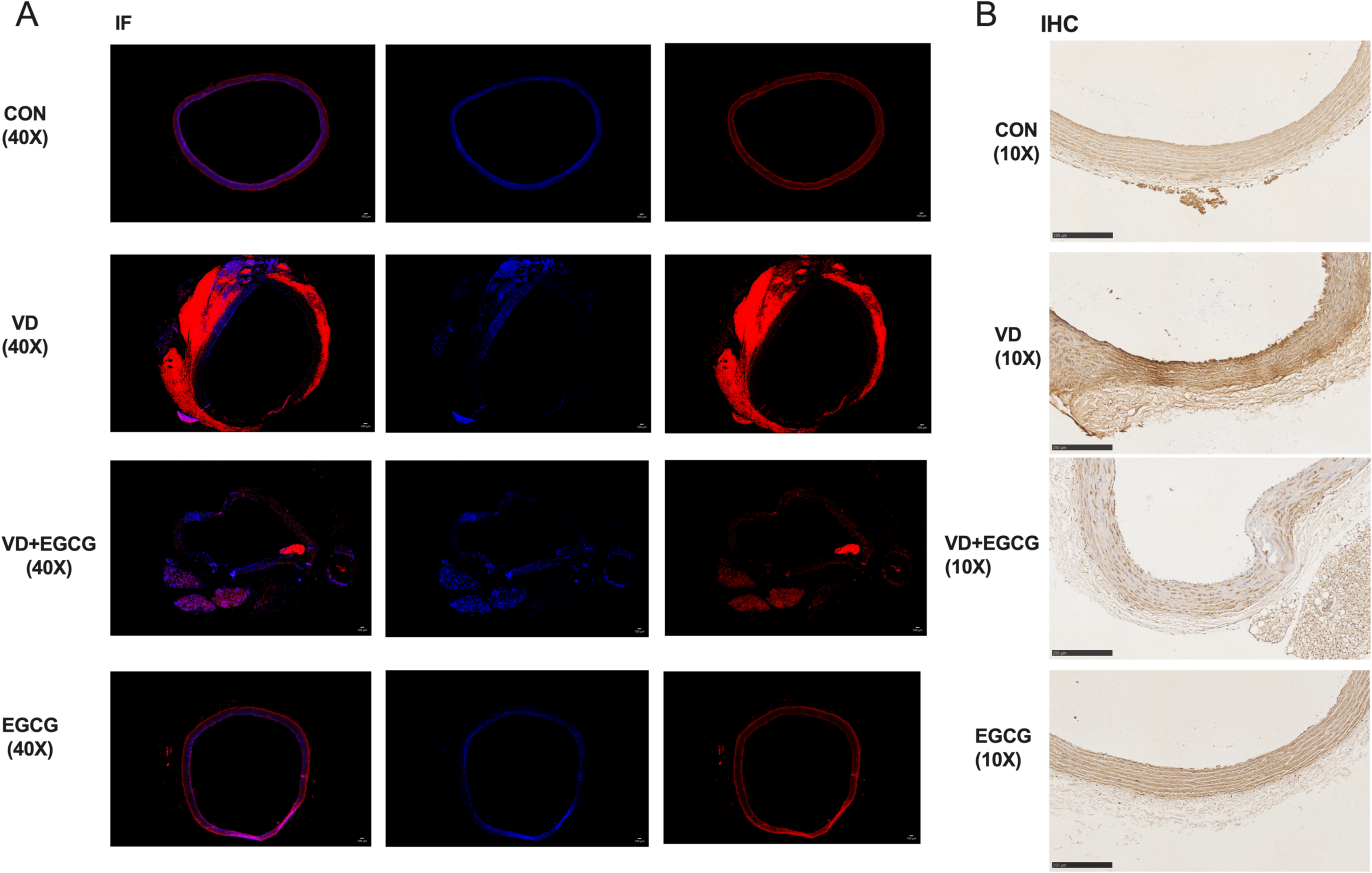
ALP immunohistochemistry and immunofluorescence results. **(A)** Immunofluorescence staining (IF) analysis of rat vascular ALP activity (magnification 40x,100μm). In immunofluorescence analysis, blue fluorescence denotes nuclear counterstaining, while red fluorescence specifically localizes alkaline phosphatase (ALP) activity. **(B)** IHC analysis of rat vascular ALP activity (magnification 10x, 250μm). Immunohistochemical staining revealed distinct chromogenic deposition patterns: hematoxylin counterstaining identified nuclei in blue, while alkaline phosphatase (ALP) activity was evidenced by brown pigment deposition, with signal intensity correlating positively with enzymatic activity.

Histological analysis of paraffin-embedded aortic sections (**Figure 2B**) and chromogen quantification (**Table 1**) confirmed elevated calcification in the VD group, with VD+EGCG treatment attenuating calcification severity. Immunohistochemical and dual-channel immunofluorescence analyses were conducted on rat aortic vessels (**Figure 3**). Immunofluorescence imaging distinguished nuclear staining (blue), ALP activity (red), and perinuclear ALP translocation (purple, co-localization of red and blue signals). **Figure 3A** revealed minimal red fluorescence in CON and EGCG groups, whereas the VD group exhibited extensive red fluorescence with limited purple co-localization, indicative of aberrant ALP activity. In contrast, the VD+EGCG group displayed reduced red fluorescence intensity and prominent purple co-localization compared to the VD group (*P<0.05), demonstrating partial normalization of ALP dynamics.

While the EGCG group showed higher red fluorescence IntDen than CON, its elevated blue fluorescence intensity suggested inter-individual vascular variability. CON and EGCG exhibited comparable Mean values, whereas VD demonstrated significantly elevated Mean and IntDen for red fluorescence versus all groups (*P<0.05). Despite increased Mean and IntDen in VD+EGCG relative to CON/EGCG, fluorescence parameters remained attenuated compared to VD, indicating therapeutic modulation.

Immunohistochemistry (**Figure 3B**) revealed ALP activity via brown chromogen deposition, with intensity proportional to enzymatic levels. CON and EGCG groups displayed faint staining, while VD showed intense deposition. VD+EGCG staining was significantly lighter than VD but did not reach CON baseline. Quantitative analysis (**Table 1**) showed equivalent Mean values between CON and EGCG, whereas VD IntDen exceeded other groups by 2.1-fold. VD+EGCG IntDen marginally surpassed CON/EGCG levels, while its Mean remained slightly lower, consistent with residual inter-individual variability. Collectively, EGCG attenuated VD-induced ALP dysregulation.

### 3.2 Epigallocatechin-3-gallate modulates gut microbiota in rats with vitamin D3-induced vascular calcification

A profound and clinically relevant connection exists between vascular calcification and gut microbiota composition. Our experimental analysis involved conducting sequencing on the 16S rRNA gene’s v3-v4 region from rat colon specimens, yielding an initial dataset of 1,623,936 raw reads across 24 biological samples. Following rigorous quality control procedures including paired-end read processing and sequence merging, we successfully obtained 1,457,849 high-confidence clean reads. The final processed data demonstrated robust sequencing depth, with each specimen maintaining a minimum of 37,886 clean reads and achieving an average yield of 66,265.86 clean reads per sample.

#### 3.2.1 Epigallocatechin-3-gallate modulates vitamin D3-induced vascular calcification in rat colon Alpha diversity and Beta diversity

**Figure 4:**
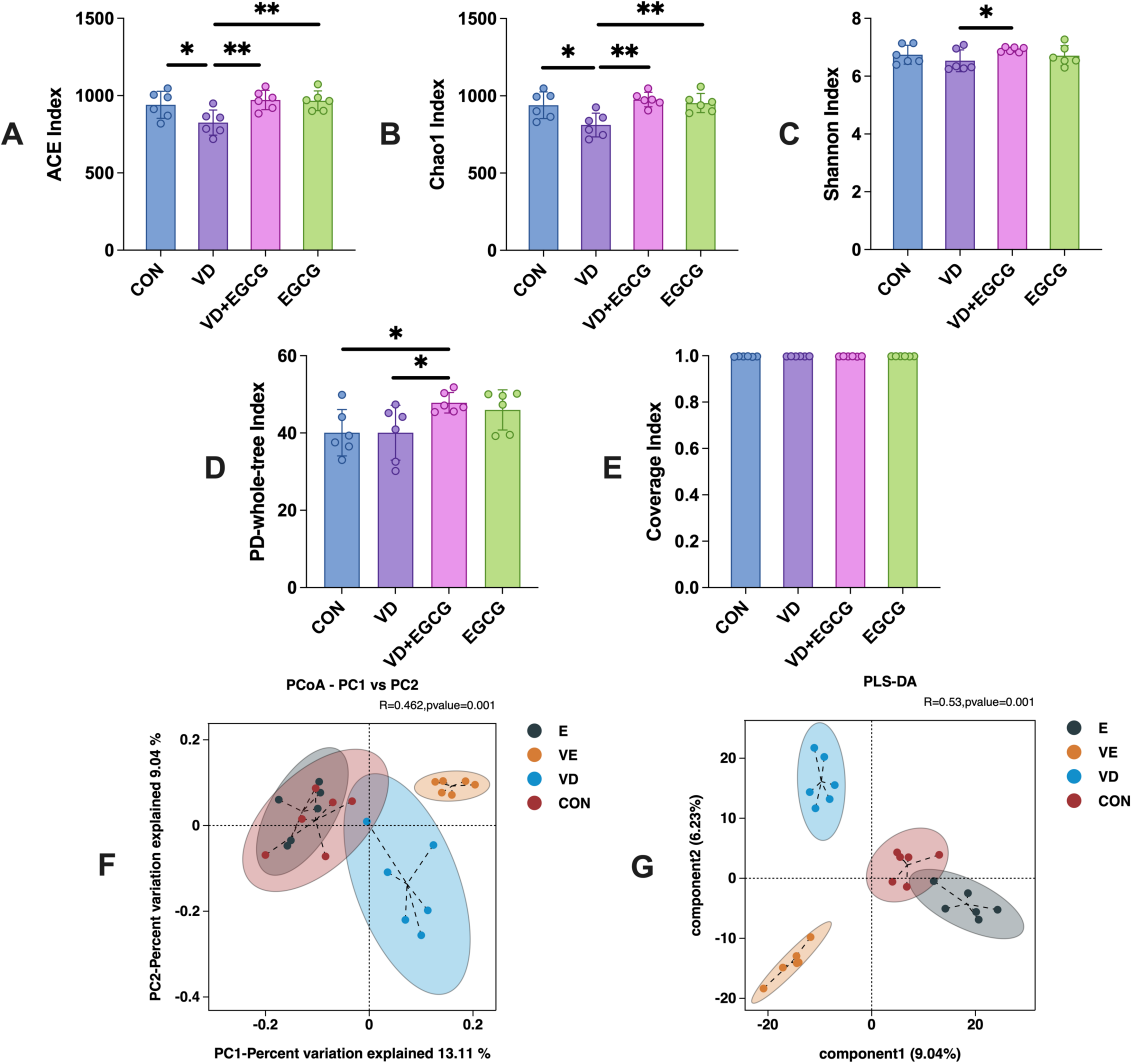
Microbial alpha and beta diversity profiles of colonic contents from 4 groups studied in rats. **(A)** ACE index, **(B)** Chao 1 index, **(C)** Shannon index, **(D)** PD whole-tree index and **(E)** coverage. Figures were analyzed by one-way ANOVA (mean ± SD). *P < 0.05 (n = 6). **(F)** PCoA plots showed that microbial community composition changed dramatically after the intervention in the VD + EGCG group (VD vs VE, *P < 0.05). **(G)** PLS-DA plots. PLS-DA plots showed significant differences between groups between group samples (*P < 0.05). Results are presented as mean ± SE *P < 0.05, determined by one-way ANOVA and Tukey’s HSD and LSD-t test. *P < 0.05; **P < 0.01.

Analysis of α-diversity revealed diminished colonic microbiota diversity in the vitamin D3 (VD)-induced vascular calcification rat model (**Figure 4**). Epigallocatechin-3-gallate (EGCG) administration effectively restored microbial richness, as evidenced by elevated ACE and PD whole-tree indices in the EGCG group compared to the CON group. Strikingly, the VD+EGCG group demonstrated significantly higher ACE, Chao 1, and PD whole-tree indices than both the VD group and the CON group, indicating enhanced microbial diversity (*P<0.05). These findings collectively suggest that oral EGCG mitigates the loss of gut microbial biodiversity triggered by vitamin D3 exposure.

Beta diversity results showed that weighted UniFrac distance from PCoA plots(*P<0.05) of out data and comparative abundance (**Figure 4F**) showed essentially constant intestinal flora in the CON and EGCG groups (*P < 0.05), but markedly different gut flora in the CON and VD groups (*P < 0.05).

Principal Coordinates Analysis (PCoA) of beta diversity revealed distinct clustering among treatment groups (**Figure 3F**), with principal coordinate 1 (PC1, 13.11%) and PC2 (9.04%) collectively explaining 22.15% of variance. While this cumulative explanatory power is modest, such values are typical for microbiome datasets due to high compositional complexity. ANOSIM analysis confirmed significant phylogenetic separation between groups (R=0.462, *P =0.001), indicating intergroup differences substantially outweighed intragroup variation.

The CON and EGCG groups exhibited near-complete overlap, demonstrating that EGCG monotherapy did not induce measurable microbiota restructuring. In contrast, pronounced divergence between VD and CON groups (*P <0.001) strongly associated VD3-induced dysbiosis with vascular calcification phenotypes. Strikingly, the VD+EGCG cluster showed no overlap with other groups, suggesting EGCG partially reversed VD3-driven microbial alterations without restoring baseline configurations. PLS-DA analysis demonstrated significant intergroup discrimination (*P < 0.05), with CON and EGCG groups showing partial overlap in the multivariate space (**Figure 4G**). Notably, Component 1 trajectories of these cohorts diverged diametrically from VD and VD+EGCG clusters, indicating EGCG monotherapy did not drive pathological shifts in healthy baseline states. Complete spatial segregation between other groups reinforced distinct intervention-specific microbial profiles.

#### 3.2.2 Epigallocatechin-3-gallate influences the number of gut microorganisms during vitamin D3 induced vascular calcification in rats

**Figure 5:**
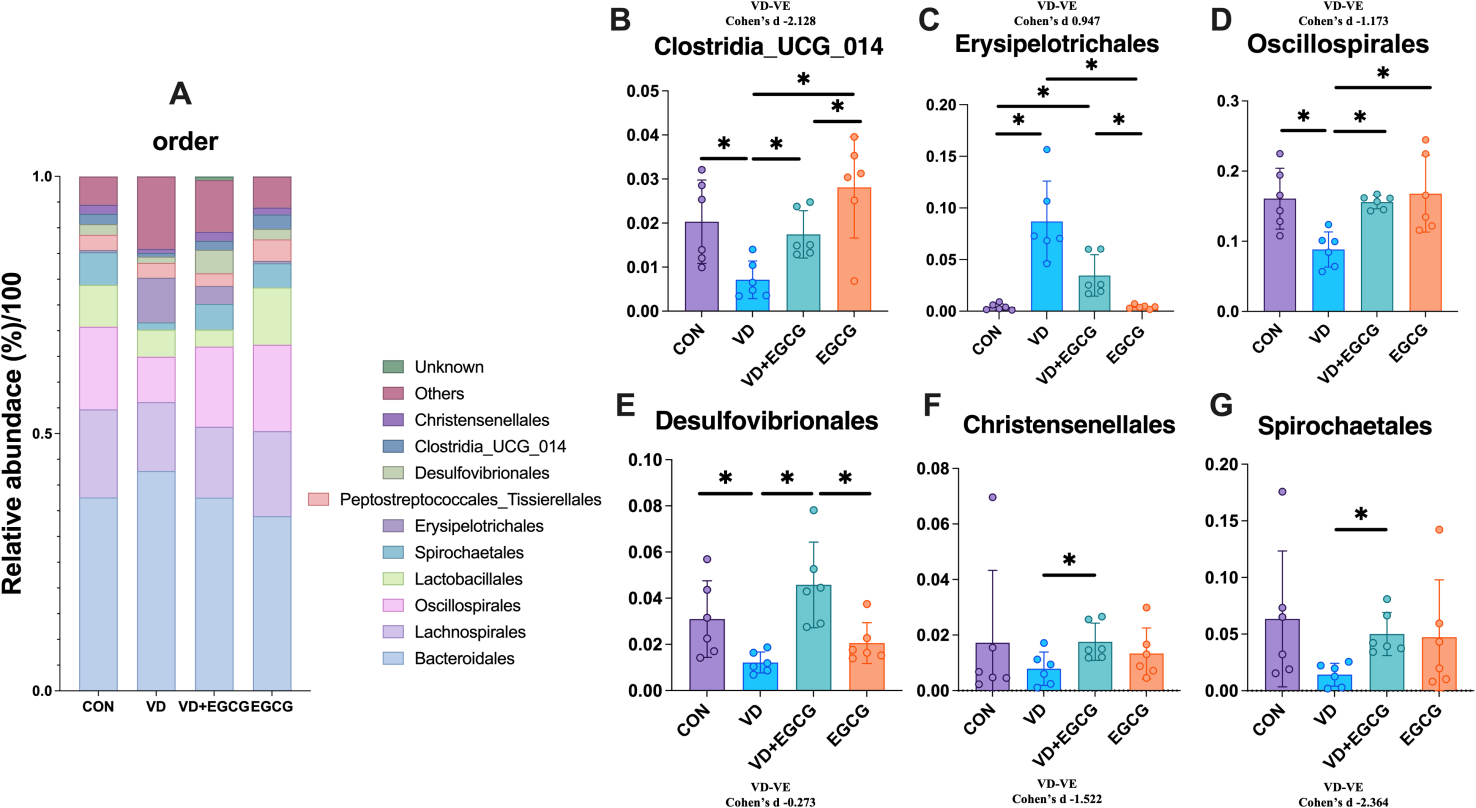
Analysis of phylum-level microbial components. **(A)** Relationship abbreviation for relative abundance of phylum-level microorganisms in rat colon. Actinobacteriota **(B)** Firmicutes **(C)** Desulfobacterota **(D)** Proteobacteria **(E)** Spirochaetota **(F)** in the colon of rats in the CON, VD, VD+EGCG and EGCG groups and Cyanobacteria **(G)** relative abundance. Results are presented as mean ± SE *P < 0.05, determined by one-way ANOVA, Tukey’s HSD, LSD-t test and Cohen’s d.

The observed fluctuations in microbial community structure and relative abundance within the gut imply a strong correlation between gut microbiota dynamics and vascular calcification pathogenesis. To delineate colonic flora characteristics in experimental rats across taxonomic hierarchies, we performed systematic taxonomic profiling of sequencing data. Dominant phyla exceeding 99% cumulative abundance included Firmicutes, Bacteroidota, Spirochaetota, Desulfobacteriota, Actinobacteriota, Proteobacteria, and Cyanobacteria (**Figure 5A**). Comparative analysis revealed significantly elevated levels of Proteobacteria and Actinobacteriota in the VD group compared to CON controls (*P < 0.05; **Figures 5B, D, F**). In contrast, Firmicutes, Spirochaetota, and Desulfobacteriota exhibited reductions of 8.75% (*P < 0.05), 5.04%, and 0.18% (*P < 0.05), respectively (**Figures 5C, D, F**), with proportional increases in Actinobacteriota and Proteobacteria (*P < 0.05; **Figures 5D, F**). EGCG administration reversed these microbial shifts, significantly reducing Actinobacteriota (*P < 0.05) and Proteobacteria levels in the VD+EGCG group post-treatment (**Figures 5B, E**). Cyanobacterial abundance varied across groups: CON (0.28%), EGCG (0.34%), VD+EGCG (0.22%), and VD (1.39%). EGCG treatment suppressed Cyanobacteria proliferation in the VD cohort, though this effect was not statistically significant.

#### 3.2.3 Epigallocatechin-3-gallate influences the number of order microorganisms in vitamin D3-induced vascular calcification in rats

**Figure 6:**
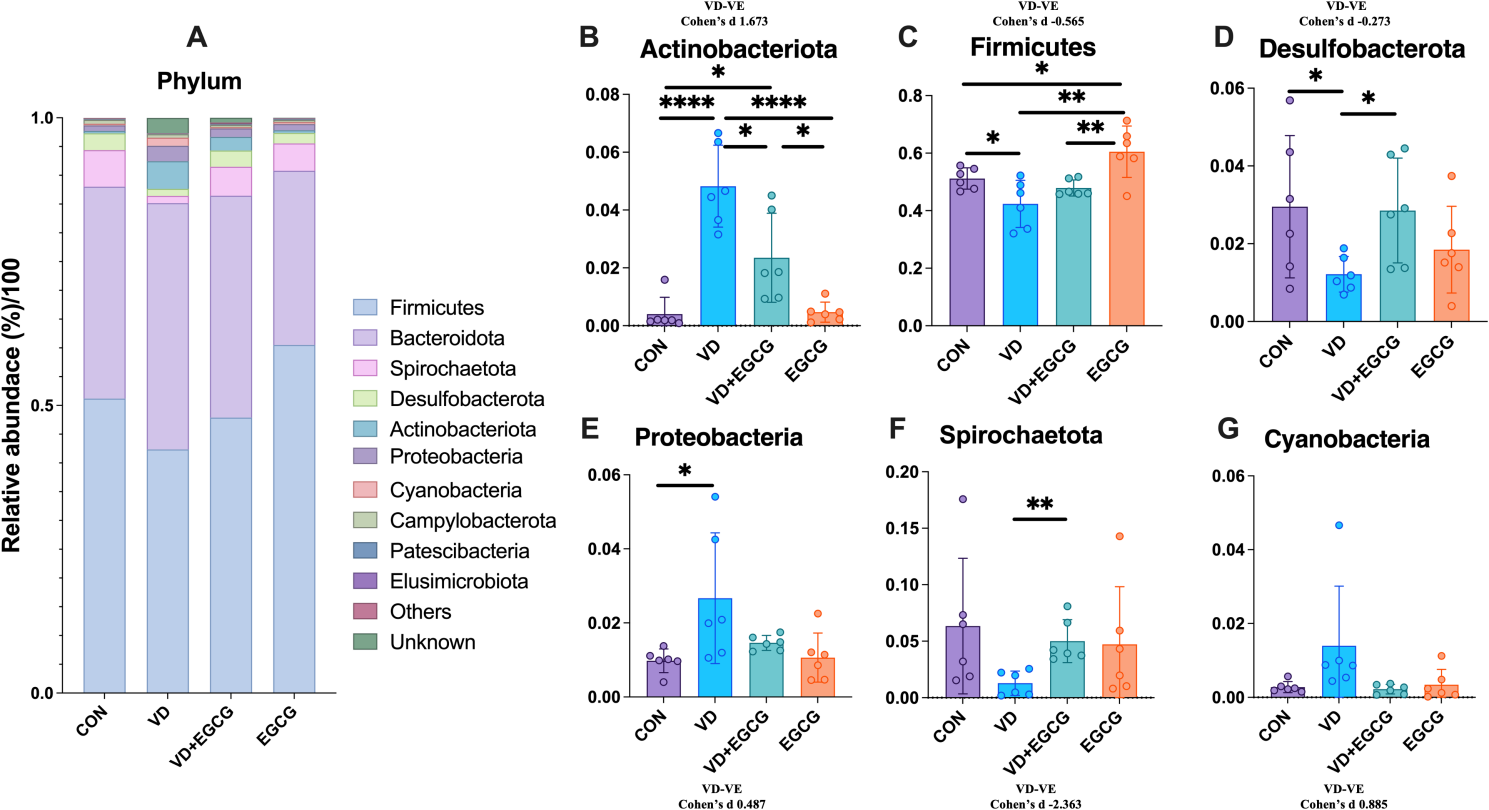
ORDER level microbial composition analysis. **(A)** Abundance of representative microbial phyla from rat colon. Clostridia_UCG_014 **(B)**, Erysipelotrichales **(C)**, Oscillospirales **(D)**, Desulfovibrionales **(E)** in the colon of rats in the CON, VD, VD+EGCG and EGCG groups, Comparison of relative abundance of Christensenellales **(F)** and Spirochaetales **(G)**. Results are presented as mean ± SE *P < 0.05, determined by one-way ANOVA, Tukey’s HSD, LSD-t test and Cohen’s d. *P < 0.05; **P < 0.01; ****P < 0.001; ns > 0.05.

The top 10 bacterial species in the contents of the rat colon are shown in **Figure 5A**. Bacteroidales, Lachnospirales, Oscillospirales, and Lactobacillales were the top four microorganisms, accounting for more than 70% of the total. Compared with the CON group, the abundance of Christensenellales, Clostridia_UCG_014, Desulfovibrionales, Spirochaetales, and Oscillospirales in the VD group decreased by 0.95%, 1.46%, 0.19%, and 4.82%, respectively, 3.11%. In addition, the abundance of Erysipelotrichales was significantly higher (*P < 0.05, **Figure 6C**). After treatment with EGCG, Christensenellales (1.75%, **Figure 6F**), Clostridia_UCG_014 (1.75%, **Figure 6B**), Desulfovibrionales (4.58%, **Figure 6E**), Spirochaetales (5%, **Figure 6G**), Oscillospirales (15.59%, **Figure 6D**) and Erysipelotrichales (3.46%, **Figure 6C**) were restored in abundance.

#### 3.2.4 Epigallocatechin-3-gallate affects the abundance of microorganisms at the level of genus and species during vitamin D3-induced vascular calcification in rats

**Figure 7:**
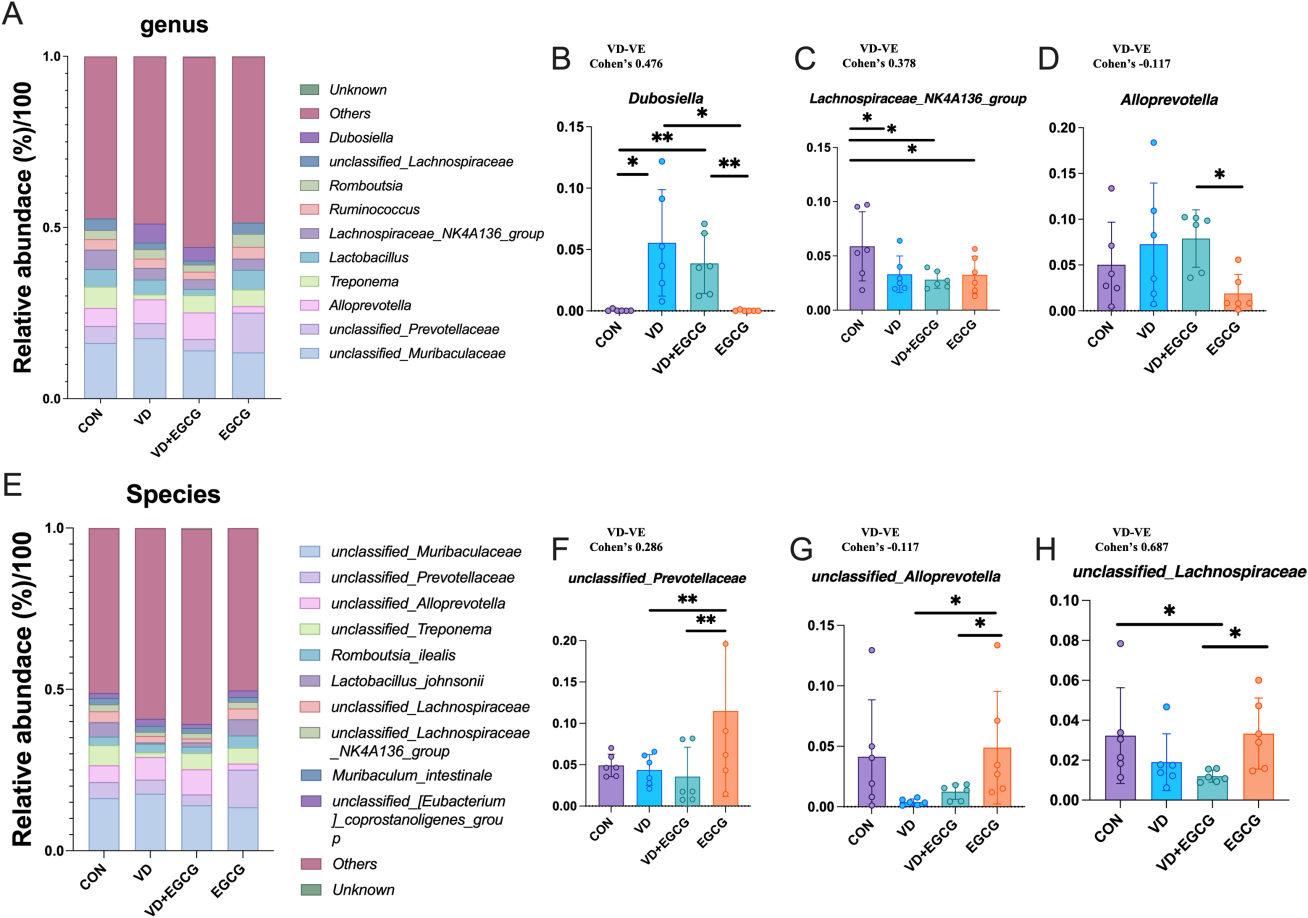
Analysis of genus and species level microscopic composition of microorganisms. **(A)** Genus-level relative abundance of microorganisms in the rat colon. Relative abundance compared of *Dubosiella* **(B)**, *Lachnospiraceae_NK4A136_group* **(C)**, and *Alloprevotella* **(D)** in the colon of rats in CON, VD, VD+EGCG, and EGCG groups. *P < 0.05 (n = 6). **(E)** Representative abundance of species levels of microorganisms in the rat colon. Relative abundance comparison of *unclassified_Prevotellaceae* **(F)**, *unclassified_Alloprevotella* **(G)** and *unclassied:Lachnospiraceae* **(H)**. Results are presented as mean ± SE *P < 0.05, determined by one-way ANOVA, Tukey’s HSD, LSD-t test and Cohen’s d. *P < 0.05; **P < 0.01.

The 10 genera and species with the highest abundance were selected for analysis (**Figures 7A, E**). *Unclassified_Muribaculaceae*, *unclassified_Prevotellaceae*, *Alloprevotella*, *Treponema*, *Lachnospiraceae_NK4A136_group*, *Ruminococcus*, *Romboutsia*, *unclassified_Lachnospiraceae*, and *Dubosiella* ranked in the top ten for abundance. The abundance of *Dubosiella* was 0.046%, 5.54%, 3.86%, and 0.023% in the CON, VD, VD+EGCG, and EGCG groups, respectively (**Figure 7B**). The data revealed that the VD group showed a marked increase in *Dubosiella* abundance, which was reversed by EGCG therapy, and the difference was more pronounced in the VD+EGCG group, but the change did not reach the level of significance (**Figure 7B**). The relative abundance of *Lachnospiraceae_NK4A136_group* was significantly reduced in the VD, VD+EGCG, and EGCG treatment groups (**Figure 7C**). *Alloprevotella* exhibited a marked increase in the VD+EGCG group but showed a significant decrease in the EGCG group; however, substantial intra-group variability precluded definitive conclusions regarding *Alloprevotella* dynamics (**Figure 7D**).

*Unclassified_Muribaculaceae*, *unclassified_Prevotellaceae*, *unclassified_Alloprevotella*, *unclassified_Treponema*, *Romboutsia_ilealis*, *Lactobacillus_johnsonii*, *unclassified_Lachnospiraceae*, *unclassified_Lachnospiraceae_NK4A136_group*, *Muribaculum_intestinale*, *unclassified_[Eubacterium]_coprostanoligenes_group*, etc. ranked in the top ten in abundance. *Unclassified_Prevotellaceae* levels fluctuated in the EGCG group, with pronounced intra-group variability obscuring potential treatment-specific effects (**Figure 7F**). Following VD3 induction, *unclassified_Alloprevotella* levels decreased by 3.99% in the VD group compared to CON, followed by a non-significant 1.1% rebound in the VD+EGCG group relative to VD (**Figure 7G**).

#### 3.3.5 Heatmap of LEfSe analysis and species abundance of colonic contents during vitamin D3-induced vascular calcification in rats affected by epigallocatechin-3-gallate

**Figure 8:**
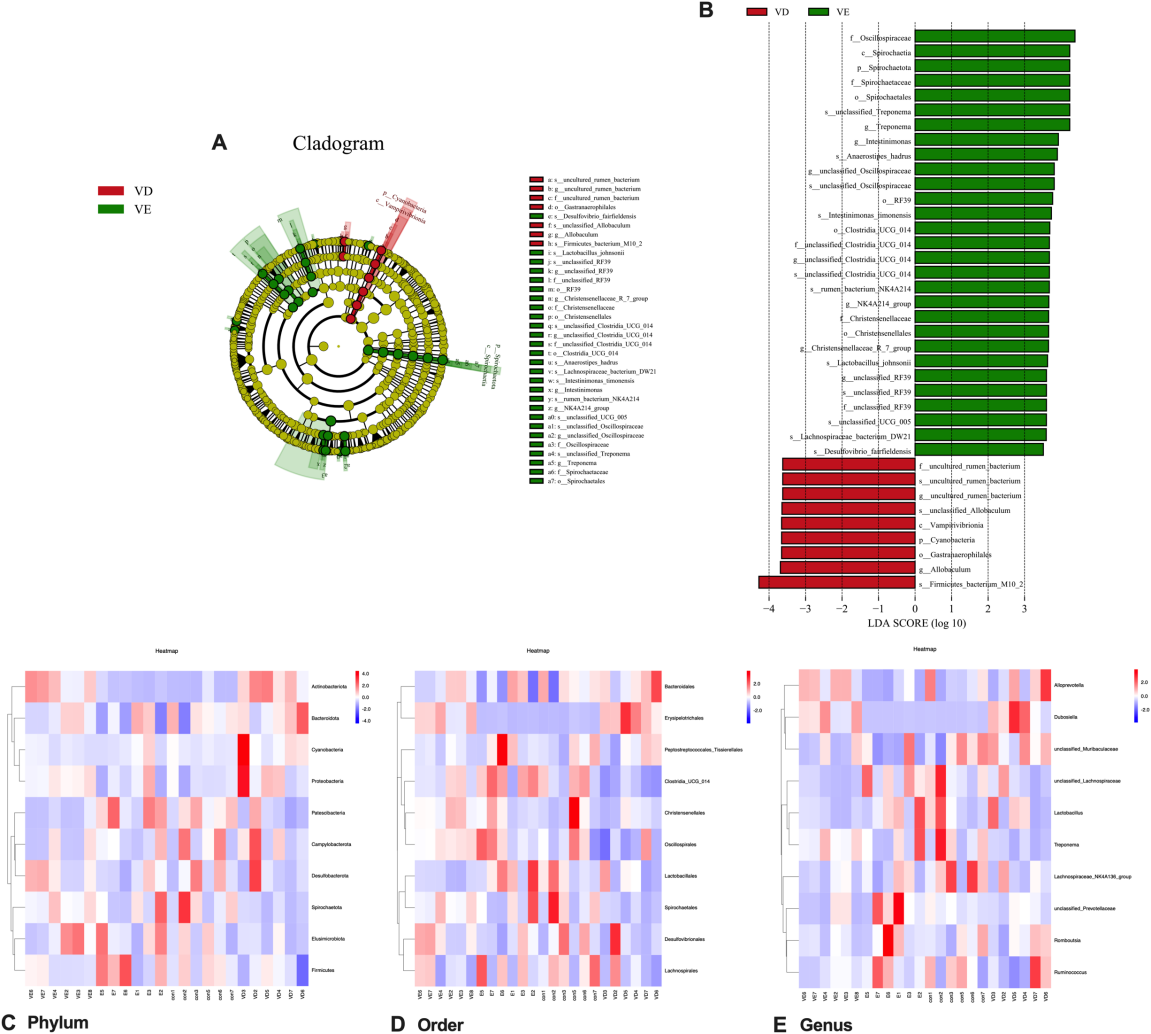
Characteristic changes of colonic contents *in vivo* in rats comparing VD and VD+EGCG groups after EGCG intervention. **(A)** Categorical representation of statistical and bioconsistency differences at baseline versus post-intervention for the VD and VD+EGCG groups. Variation is indicated by the richest category color (red at baseline, green after intervention). Each circle’s diameter is proportional to the richness of the categorical unit. **(B)** Bar chart of linear discriminant analysis (LDA) points calculated for traits of different richness in the VD and VD+EGCG groups. The horizontal line indicates the effect magnitude for each classification. Column lengths indicate log10-transformed LDA scores. The threshold for log LDA scores for discriminant traits was set at 3.5. **(C)** Heatmap of the top 10 gut microbial phyla by relative abundance. **(D)** Heatmap of the top 10 gut microbial orders by relative abundance. **(E)** Heatmap of the top 10 gut microbial genera by relative abundance. Color scale corresponds to Z-values of normalized relative abundance.

LEfSe analysis results of colonic microorganisms in the VD and VD+EGCG groups are displayed (*P < 0.05, **Figure 8A**). Significantly abundant in the VD group were Oscillospiraceae, Spirochaetia, Spirochaetota, Spirochaetaceae, Spirochaetales in the VD group, *unclassified_Treponema*, *Treponema*, *Intestinimonas*, *Anaerostipes_hadrus*, *unclassified_Oscillospiraceae*, RF39, *Intestinimonas _fimonensis*, Clostridia_UCG_014, *unclassified Clostridia_UCG_014*, *rumen_bacterium_NK4A214*, *NK4A214_group*, Christensenellaceae, Christehsenellales, *Christensenellaceae_R_7_group*, *Lactobacillus johnsonii*, *unclassified RF39*, *unclassified_UCG_005*, *Lachnospiraceae_bacterium_DW21*, *Desulfovibrio _fairfieldensis*. Significantly abundant in the VD+EGCG group were *uncultured_rumen_bacterium*, *unclassified_Allobaculum*, Vampirivibrionia, Cyanobacteria, Gastranaerophilales, *Allobaculum*, *Firmicutes_bacterium M10_2*. Linear discriminant analysis (LDA) was employed to measure effects of species richness on between-group differences and indicated that all species had LDA scores above 3, with *Firmicutes_bacterium M10_2* in the VD group having an LDA score above 4 (**Figure 8B**). Heatmaps of gut microbiota composition at the phylum, order, and genus levels (**Figures 8C-E**) demonstrate a compositional shift that was attenuated in the EGCG intervention group.

### 3.3 Effects of epigallocatechin-3-gallate on serum concentration of total metabolites in rats

**Figure 9:**
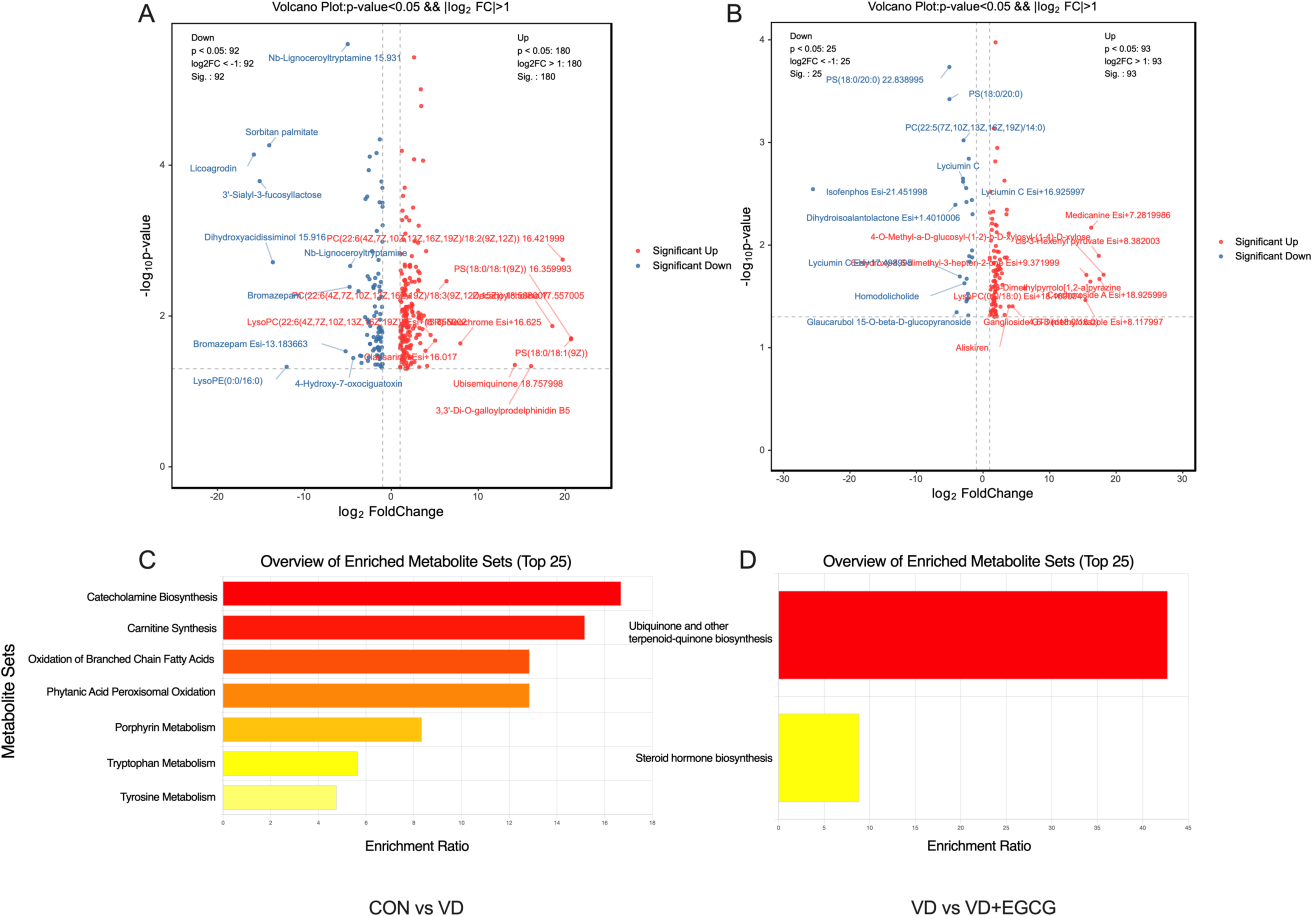
Results of differentiated metabolite and KEGG pathway enrichment analyses. **(A)** Volcanogram analysis of the differentiated metabolites from CON and VD; **(B)** Volcanogram analysis of the differentiated metabolites from VD and VD+EGCG; **(C)** Results of enrichment analysis of KEGG pathways from CON and VD; **(D)** Results of enrichment analysis of KEGG pathways from VD and VD+EGCG.

Relevant serum metabolites were quantified in rats using LC-MS (**Figures 9A, B**). Pairwise comparisons were comprehensively analyzed for serum differential metabolites in the CON, VD and VD+EGCG groups of SD rats. Notably, metabolites with P values <0.05, log2 values >1 and log2 values <-1 were considered significant. Differential metabolite volcano plots showed a total of 272 unique metabolites between the CON and VD groups (**Figures 9A, B**). Among them, PC (22:6(4Z,7Z,10Z,13Z,16Z,19Z)/18:2(9Z,12Z)) 16.421999, PS (18:0/18:1(9Z)) 16.359993, Ubisemiquinone 18.757998, Decanoylcholine 17.557005, PC(22:6(4Z,7Z,10Z,13Z,16Z,19Z)/18:2(9Z,12Z)) 21.041994, (8’R)-Neochrome Esi+16.625, Ubisemiquinone 18.757998,3,3’-Di-O-galloylprodelphinidin B5, LysoPC(22:6(4Z,7Z,10Z,13Z,16Z,19Z)) Esi+15.855002, and Clausarinol Esi+16.017 were up-regulated in the VD group, while Nb-Lignoceroyltryptamine 15.931, Sorbitan palmitate, Licoagrodin, 3’-Sialyl-3-fucosyllactose, Dihydroxyacidissiminol 15.916, Nb-Lignoceroyltryptamine, Bromazepam, Bromazepam Esi-13.183663, LysoPE (0:0/16:0), and 4-Hydroxy-7-oxociguatoxin expression were downregulated. On the other hand, 84 distinct metabolites were identifiable among the VD and VD+EGCG groups. After oral administration of EGCG, including medicanine Esi+7.2819986, 4-O-Methyl-a-D-glucosyl-(1-2)-b-D-xylosyl-(1-4)-D-xylose, cis-3-Hexenyl pyruvate Esi+8.382003, Ganglioside GT3 (d18:0/18:0), Aliskiren, 4,5-Dimethyloxazole Esi+8.117997, 1,3-Dimethylpyrrolo[1,2-a]pyrazine exhibited significant up-regulation, whereas Medicane Esi+ 7.2819986, 4-O-Methyl-a-D-glucosyl-(1-2)-b-D-xylosyl-(1-4)-D-xylose, cis-3-Hexenyl pyruvate Esi+8.382003, Ganglioside GT3 (d18:0/18:0), Aliskiren, 4,5-Dimethyloxazole Esi+8.117997, 1,3-Dimethylpyrrolo[1,2-a]pyrazine, Lyciumin C, PS (18:0/20:0) 22.838995, PS (18:0/20:0), Isofenphos Esi-21.451998 showed reduced expression.

Notably, Ubisemiquinone has a role in blocking phenotypic transformation and aortic degeneration in cardiovascular disease, Aliskiren has a role in vascular remodeling and improving vascular smooth muscle function in cardiovascular disease, and Bromazepam has a thrombolytic role in cardiovascular disease.

Functional profiling of major differential metabolites through Kyoto Encyclopedia of Genes and Genomes (KEGG) database concentration analysis (**Figures 9C, D**) revealed distinct metabolic pathway divergences. Comparative evaluation between CON and VD groups identified significant variations in catecholamine biosynthesis, carnitine synthesis, branched chain fatty acid oxidation, phytanic acid peroxisomal oxidation, porphyrin metabolism, tryptophan metabolism, and tyrosine metabolism. Notably, the VD versus VD+EGCG comparison demonstrated marked alterations in ubiquinone/terpenoid-quinone biosynthesis and steroid hormone biosynthesis pathways. These metabolic alterations offer critical mechanistic links to cardiovascular pathogenesis while highlighting EGCG’s substantial regulatory capacity through targeted intervention.

### 3.4 Correlation of gut microorganisms with serum metabolites

**Figure 10:**
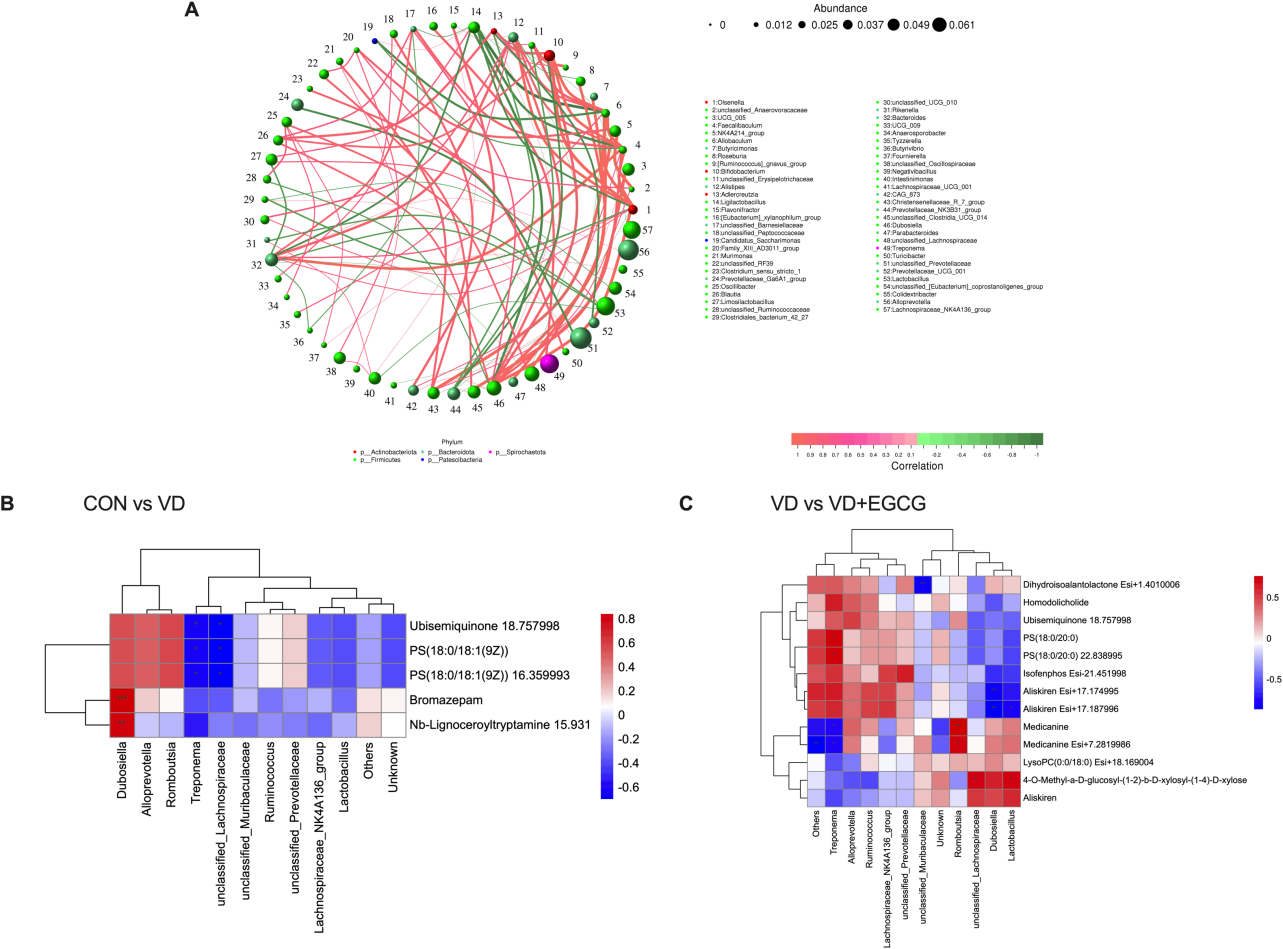
Spearman correlation analysis of intestinal microbiota-serum metabolite interactions. **(A)** Microbial co-occurrence network topology in rat gastrointestinal ecosystems. **(B)** Hierarchical clustering analysis of associations between gut microbiota differentials and serum metabolic profiles in CON-VD group comparisons. **(C)** Matrix visualization of microbe-metabolite covariation patterns specific to VD versus VD+EGCG interventions.

The Spearman correlation analysis of genus-level networks of gut flora in all groups of rats (**Figure 10A**) indicated that LIgliactopacilus, Candidatus_Saccharimonas, Prevotellaceae_Ga6A1_group, Prevotellaceae_NK3B31_group, unclassified_Prevotellaceae, and Prevotellaceae_UCG_001 were highly negatively correlated with other microorganisms, while unclassified Anaerovoracaceae, UCG_005, aecalibaculum, and Allobaculum, NK4A214_group, Bifidobacterium, Alistipes, unclassified_Barnesiellaceae, Blautia, Bacteriodes, Christensenellaceae_R_7_group, Dubosiella, unclassified _Lachnospiraceae were highly positively correlated with other microorganisms.

Heatmaps of correlations between gut microbes and serum differential metabolites showed (**Figures 10B, C**) that in the correlations between the CON and VD groups (**Figure 10B**), *Dubosiella* and all other metabolites were positively correlated, with significance with Bromazepam and Nb-Lignoceroyltryptamine 15.931. *Romboutsia*, *Alloprevotella* were positively correlated with other metabolites except for a negative correlation with Nb-Lignoceroyltryptamine. Two microorganisms, *Treponema* and *unclassifie_Lachnospiraceae*, were positively correlated with the metabolites Ubisemiquinone 18.757998, PS (18:0/18:1 (9Z)), and PS (18:0/18:1 (9Z)) 16.359993 were significantly negatively correlated. In the VD and VD+EGCG groups (**Figure 10C**), *unclassified_Muribaculcaceae* and Dihydroisoalantolactone Esi+1.4010006 had a significant negative correlation. *Alloprevotella* and the other metabolites showed a positive correlation, except for 4-O- Methyl-a-D-glucosyl-(1-2)-b-D-xylosyl-(1-4)-D-xylose, Aliskiren and LysoPC (0:0/18:0) were negatively correlated. In *Treponema* microorganisms showed significant positive correlation with PS(18:0/20:0) and PS(18:0/20:0) 22.838995 and significant negative correlation with Medicanine and Medicanine Esi+7.2819986, microorganisms *Romboutsia* showed significant positive correlation with metabolites Medicanine and Medicanine Esi+7.2819986 were significantly positively correlated, and microorganisms *unclassified_Lachnospiraceae* and *Lactobacillus* were significantly positively correlated with metabolites 4-O-Methyl-a-D-glucosyl-(1-2)-b-D-xylosyl-(1-4)-D- xylose also showed significant positive correlation.

## 4 Discussion

Vascular calcification, a clinical manifestation of cardiovascular disease, is strongly associated with cardiovascular mortality and represents a major global health threat. Emerging evidence suggests that probiotics may ameliorate cardiovascular pathologies by modulating gut microbiota composition and preserving intestinal barrier integrity. Epigallocatechin-3-gallate (EGCG), a bioactive plant polyphenol, acts as a potent prebiotic that regulates gut microbiota while exhibiting anti-inflammatory, antioxidant, and anticancer properties. This study investigates the mechanisms underlying EGCG-mediated attenuation of vascular calcification in rats. Serum metabolomic and gut microbiome analyses revealed that EGCG exerts therapeutic effects on vascular calcification via gut microbiota-dependent pathways.

Alizarin red S staining (which binds calcium ions for histochemical quantification) demonstrated markedly reduced calcium deposition in the EGCG-treated group compared to the model group, as evidenced by both colorimetric contrast and fluorescence quantification. Notably, while body weight trends in the VD+EGCG group paralleled those in the VD group (potentially reflecting inter-individual dietary variability), EGCG intervention significantly attenuated vascular calcification. Although the EGCG group exhibited a more pronounced increase in food intake compared to the CON group, the precise biological significance of this effect remains unclear. Due to the substantial error margins in **Figure 1**, it is not possible to definitively establish a causal relationship with EGCG consumption. Immunohistochemistry (IHC) and immunofluorescence (IF) analyses confirmed that EGCG reduced calcific deposits and suppressed alkaline phosphatase (ALP) activity, a key mediator of mineralization. Quantitative comparisons revealed that ALP activity in the treatment group remained elevated relative to control (CON) and EGCG-alone groups, yet significantly lower than in VD models, underscoring the link between ALP modulation and calcification reduction. These findings collectively demonstrate EGCG’s therapeutic potential in mitigating vascular calcification.

Sequencing of colonic microorganisms revealed that EGCG dramatically modulated the architecture of the rat intestinal microbial communities, with a marked percentage gain in the proportion of beneficial flora, and that two intestinal microorganisms, Christensenellales and Oscillospirales, are known to be probiotic, whereas other microorganisms had significantly increased abundance in the treated group but there is not yet enough information to indicate that they are probiotics. Christensenellales are thought to be part of the gut microbial community that may influence host metabolism. Christensenellaceae minuta, as a specific microorganism, may play a role in the regulation of host metabolism and weight gain by modulating changes in the gut flora of the host (20), and one of the major pathways regulating metabolites is the Wnt signaling pathway, which is a major pathway known to regulate vascular calcification. Christian Freise et al. (21) demonstrated that the Wnt signaling pathway was an important regulator of vascular calcification by examining whether MMP-mediated VSMCs calcification involved alterations in Wnt pathway signaling, which was known to affect osteogenesis. In Christian’s study, Al-Aly Z described a novel TNF-α-regulated Msx2-Wnt osteogenic program that regulated arterial calcification in a type 2 DM animal model (22). Therefore, we hypothesized that Christensenellales alleviated vascular calcification in rats by modulating the Wnt signaling pathway. Oscillospirales are producers of butyrate (23), butyrate is a type of SCFAs, and a study by Yan, J. et al. was conducted through vitamin D3 and nicotine (VDN). In a vitamin D3 and nicotine (VDN)-induced vascular calcification model in rats, oral or rectal administration of propionate remodeled the gut microbiota, which resulted in an increase in the production of SCFA, an improvement in intestinal barrier function, a reduction in inflammation, and eventually an amelioration of vascular calcification, and the results indicated that SCFA (i.e., propionate and butyrate) were isolated protecting factors that inhibited vascular calcification (24). Thus, EGCG is used to treat vascular calcification by elevating the abundance of Oscillospirales and thereby modulating SCFAs in the gut. Actinobacteriota, Erysipelotrichales contains a number of Gram-positive bacteria that cause infections when the immune system is compromised, and the bacteria in Proteobacteria, Cyanobacteria include a number of pathogens. Actinobacteriota possess phospholipase activity capable of generating the pro-inflammatory metabolite trimethylamine (TMA) through phosphatidylcholine (PC) catabolism. In contrast, the order Daniofilariae plays a central role in choline metabolism, where CutC/D enzymes convert choline to TMA (25). Subsequent oxidation of TMA yields trimethylamine N-oxide (TMAO), a metabolite associated with increased vascular calcification risk. Proteobacteria, Cyanobacteria also include several pathogens, and *Dubosiella* is a Gram-negative, non-spore-forming, exclusively anaerobic group of bacteria belonging to the Bacteroidetes. The bacteria of *Dubosiella* are usually found in a symbiotic relationship with the intestinal tracts of humans and animals. However, *Dubosiella* may also cause intestinal disorders under certain specific circumstances, especially if the immune system is compromised or the intestinal barrier function is impaired. However, there is no evidence directly linking these bacteria to vascular calcification. Therefore, it is hypothesized that *Dubosiella*, Erysipelotrichales, Cyanobacteria, Proteobacteria, and Actinobacteriota, which are in the VD group and show a significant increase in abundance, contribute to the worsening of vascular calcification diseases.

Serum metabolomic profiling revealed significant perturbations in multiple pathways, including catecholamine biosynthesis, carnitine synthesis, branched chain fatty acid (BCFA) oxidation, phytanic acid peroxisomal oxidation, porphyrin metabolism, tryptophan metabolism, and tyrosine metabolism. Substantial alterations were also observed in ubiquinone/terpenoid-quinone biosynthesis and steroid hormone biosynthesis pathways. Of particular interest, BCFA oxidation — a process mechanistically associated with energy homeostasis (26)— demonstrated no established direct correlation with vascular calcification pathways in current literature. Nevertheless, broader fatty acid metabolic regulation has been conclusively linked to the phenotypic differentiation of vascular smooth muscle cells (VSMCs). Glycolysis and fatty acid *de novo* synthesis pathways with fatty acid synthase FASN-mediated palmitate generation synergistically promote dedifferentiation and neointimal hyperplasia in VSMCs. Schiattarella et al. demonstrated that trimethylamine-N-oxide, a metabolite produced by gut microorganisms, acts as a biomarker for cardiovascular risk (27), Chen et al. demonstrated that resveratrol modulates TMAO synthesis and bile acid metabolism through remodeling of the gut microbiota and attenuates TMAO-induced atherosclerosis (28), and Seldin et al. demonstrated that TMAO promotes vascular inflammation through MAPK and NF-κB signaling (29). And in the study of Liliana C. Baptista et al. it was mentioned that the metabolomic analysis of patients with cardiovascular disease under the intervention of resveratrol, found Tryptophan Metabolism in relation to TMAO and cardiovascular disease (30). Resveratrol and EGCG are both plant-based polyphenols with very similar anti-inflammatory and antioxidant properties. By analogy, EGCG can also improve cardiovascular disease by mediating Tryptophan Metabolism, and abnormal Tryptophan Metabolism will lead to abnormal serotonin levels, and excessive serotonin will affect the function of osteoblasts and osteoclasts, which will mediate the expression of bone metabolism-related factors, such as bone morphogenetic protein-2, to promote the calcification of VSMCs. promote calcification of VSMCs. Tryptophan Metabolism produces intermediates that are cytotoxic such as 3-hydroxykynurenine, which are intermediate metabolites that can damage the vascular endothelial cells, disrupting their integrity and exposing the cells to substances in the blood, such as Ca and P, which in turn can lead to calcification. Tryptophan Metabolism is in turn related to nutrient metabolism, where a vitamin B6 Deficiency leads to the cumulative production of pyridoxal phosphate, which in turn disrupts the dynamic balance of mineral metabolism such as Ca, P, etc., allowing calcium and phosphorus salt deposits to be deposited in the vessel wall, leading to vascular calcification. Certain factors released from calcified blood vessels may inhibit the activity of tyrosine hydroxylase, blocking the conversion of tyrosine to catecholamines, leading to changes in the levels of tyrosine and its metabolites in the body, further affecting the physiological functions of the body. This process affects Tyrosine Metabolism and Catecholamine Biosynthesis. Ubiquinone (coenzyme Q10) is an important product of Ubiquinone and other terpenoid-quinone biosynthesis, which scavenges free radicals produced in the cell, thereby reducing the effects of oxidative stress. When the level of oxidative stress is too high, it can damage vascular endothelial cells and promote vascular calcification. An increase in coenzyme Q10 enhances the antioxidant defense system and has an inhibitory effect on vascular calcification. The metabolites produced by this pathway act as electron transfer through the mitochondrial respiratory chain and are essential for the synthesis of ATP, the energy required for cellular physiological activity. VSMCs play an important role in resisting calcification during vascular calcification, and the energy required by VSMCs comes mainly from this pathway. Abnormalities in Ubiquinone and other terpenoid-quinone biosynthesis and problems with energy supply can lead to dysfunction of vascular smooth muscle cells and a phenotypic shift of VSMCs to osteoblast-like cells, which promotes vascular calcification. Steroid hormone biosynthesis Cholesterol produced in the pathway is converted to vitamin D. Activated vitamin D permeates the bloodstream as a hormone, and excess can lead to elevated blood calcium, increasing the risk of vascular calcification. Other metabolites have no direct pathways or metabolic routes that have been shown to be associated with vascular calcification.

Plant polyphenols exhibit concentration-dependent biological effects, with epigallocatechin-3-gallate (EGCG) demonstrating multifunctional activity. At therapeutic doses, EGCG acts as a potent anti-inflammatory, anticancer, and antioxidant agent, scavenging reactive oxygen species (ROS) and suppressing immune-mediated autophagy. While Riegsecker et al. (31) reported EGCG’s efficacy in mitigating vascular inflammation, studies by Liu et al. (32), Wang et al. (33), and Tipoe et al. (34) identified hepatotoxic risks at elevated doses, including liver injury induced by galactosamine, amantadine A, and carbon tetrachloride in rodent models (35). These findings underscore the critical challenge of optimizing EGCG dosage to balance efficacy and safety.

A conservative dose of 50 mg/kg/day has been widely employed in preclinical studies. For example, Zhenhua Wu et al. (36) demonstrated that 50 mg/kg/day EGCG attenuated dextran sulfate sodium (DSS)-induced colitis in inflammatory bowel disease (IBD) models by enhancing short-chain fatty acid (SCFA) production via Akkermansia and other SCFA-producing gut microbiota. Similarly, Yi Cai et al. (11) showed that 50 mg/kg/day EGCG inhibited pressure overload-induced cardiac hypertrophy through modulation of the PSMB5/Nmnat2/SIRT6 signaling pathway. Higher doses, however, may enhance therapeutic outcomes: Rui Sheng et al. (37) reported that 50 and 100 mg/kg EGCG reduced c-Myc protein levels to 78.3% and 64.8%, respectively, in aortic stenosis models, while telomere repeat binding factor 2 (TRF2) levels decreased to 65.2% (50 mg/kg) and 87.1% (100 mg/kg) compared to controls (P < 0.01). Both doses also suppressed p53 protein expression (66.0% and 63.8% of baseline, respectively), confirming EGCG’s role in mitigating telomere shortening and cardiomyocyte apoptosis, with 100 mg/kg exhibiting superior efficacy. Supporting this, Haichao Deng et al. (38) identified 109.81 mg/kg as the optimal dietary EGCG dose for Monopterus albus (Chinese rice eel). Given these findings, we selected 100 mg/kg/day for our experimental design, hypothesizing enhanced therapeutic benefits without hepatotoxicity at this dose.

Our previous work demonstrated that epigallocatechin-3-gallate (EGCG) ameliorates vitamin D3 (VD3)-induced vascular calcification. Emerging evidence suggests EGCG may also attenuate calcification in alternative models. For instance, in chronic kidney disease (CKD) with medial arterial calcification (MAC), Tiantian Li et al. (39) reported that EGCG dose-dependently reduced calcium phosphate deposition and vascular smooth muscle cell (VSMC) osteogenic differentiation both *in vivo* and *in vitro*, suppressing MAC in a 5/6 nephrectomy model via JunB-dependent inhibition of the Ras/Raf/MEK/ERK signaling pathway.

Nhung Thi Nguyen et al. (40) investigated molecular mechanisms linking phosphate (Pi)-induced oxidative stress to intracellular calcium dysregulation. Their work revealed that elevated extracellular Pi upregulated protein expression and membrane trafficking of Pi transporters PiT1 and PiT2 in VSMCs, establishing a direct relationship between calcium (Ca^2+^), phosphate, and vascular calcification. Notably, multiple studies have shown that EGCG modulates calcium channels to regulate intracellular Ca^2+^ concentrations (41, 42). While no direct studies have tested EGCG in adenine- or calcium diet-induced vascular calcification models, its established regulatory effects on Pi and Ca^2+^ homeostasis suggest therapeutic potential in these contexts.

In this article, we focused on the therapeutic effect of EGCG on vitamin D3-induced vascular calcification in rats. The data of colonic microbial and metabolite level assays proved the therapeutic effect of EGCG on vascular calcification, but the low bioavailability of EGCG and its low solubility is a great challenge. In our study, many microorganisms with significant differences in abundance, as well as differential metabolites and metabolic pathways, which had not been seen in previous studies, were identified. More studies are needed in the future to thoroughly and comprehensively investigate the exact mechanism of relieving vascular calcification, the therapeutic dose of EGCG, whether EGCG is toxic at high doses, and the range of doses used for treating vascular calcification, which will help to maximize efficacy in controlling vascular calcification and to explore a new avenue for treating vascular calcification.

An 11-week EGCG intervention in Sprague-Dawley (SD) rats revealed that EGCG ameliorates vascular calcification. However, the persistence of EGCG-induced gut microbiota restructuring and metabolite alterations post-cessation remains unclear. EGCG administration increased beneficial taxa abundance in the gut microbiota irrespective of treatment duration (short- or long-term). We hypothesize that post-intervention dietary maintenance may sustain microbiota composition and beneficial taxa dominance.

The concept of caloric restriction mimetics (CRMs)—pharmacologically active compounds that recapitulate the multifaceted effects of caloric restriction (CR)—has recently emerged (43–47). CRMs enhance autophagy, a process whose age-associated decline contributes to progressive tissue deterioration and is implicated in neurodegenerative and cardiovascular pathologies (48–50). Despite their therapeutic potential, clinically validated CRM candidates remain scarce.

Epicatechin and epigallocatechin-3-gallate (EGCG), flavan-3-ol subclass flavonoids, exhibit cardiometabolic benefits. However, clinical trials of EGCG have predominantly targeted neurodegenerative diseases and cancer (51, 52), with limited investigation into cardiovascular applications. In contrast, Zhen Zhang et al. (53) revealed that the antihypertensive effect of human induced pluripotent stem cell-derived mesenchymal stromal cells (hiPSC-MSCs) was abolished following splanchnic nerve denervation (SND), highlighting the splanchnic nerve’s essential role. Splanchnic nerve activation elevated norepinephrine (NE) release, which increased splenic and peripheral blood choline acetyltransferase-positive (ChAT^+^) cell populations. The consequent rise in acetylcholine (ACh) production mediated vasodilation, reducing blood pressure and inflammatory responses in end organs. This mechanistic interplay between neural signaling and vascular pathophysiology informs translational strategies for cardiovascular disease trials.

A translational gap persists between preclinical (*in vitro*/*in vivo*) and clinical studies of epigallocatechin-3-gallate (EGCG) in disease treatment, potentially attributable to its limited bioavailability—particularly in the brain. The low oral bioavailability of EGCG remains a major pharmacokinetic challenge. While cellular experiments typically employ EGCG concentrations of 10–100 μM, oral administration of 3 g decaffeinated green tea yields a plasma Cmax of only 0.7 μM. Notably, escalating the dose to 4.5 g failed to significantly elevate Cmax, indicative of absorption saturation. For example, a clinical trial administering 800 mg/day EGCG to prostate cancer patients for 3–6 weeks pre-surgery detected no EGCG in resected tissues, underscoring bioavailability constraints (54).

Recent advances in drug delivery systems aim to overcome these limitations. Strategies such as Pickering emulsions (55, 56), liposomal/nanosomal encapsulation (57), and bilosomes—vesicles embedded with bile salts—have enhanced EGCG stability and bioavailability. Bilosome-based delivery, for instance, increased EGCG bioavailability by 1.98-fold (58). These innovations address key barriers to EGCG’s therapeutic translation.

Human-animal disparities pose a major translational challenge for epigallocatechin-3-gallate (EGCG) clinical trials. Osteopontin splice variant (OSP), a phosphorylated glycoprotein critical for bone development, contains a conserved Arg-Gly-Asp (RGD) cell-binding sequence that mediates calcium binding, chemotaxis, and adhesion in rat vascular smooth muscle cells (VSMCs) (59). OSP expression—at both transcriptional and translational levels—is regulated by alkaline phosphatase (ALP) activity, which generates extracellular inorganic phosphate (Pi). This Pi signaling is essential for OSP induction, forming a feedback loop: ALP activity increases Pi release, while Pi subsequently inhibits ALP (60). Despite OSP’s strong association with vascular calcification *in vivo*, studies demonstrate minimal OSP expression in human VSMCs compared to rodent counterparts, highlighting species-specific differences in calcification biology (59). Further interspecies divergence exists in catechin metabolism: while animal models employ supraphysiological EGCG doses, humans typically ingest small quantities via dietary sources (e.g., ~50–100 mg per tea cup).

Current barriers to translating EGCG into vascular calcification trials include its low oral bioavailability, unoptimized delivery systems, and insufficient validation of human dosing regimens. These factors necessitate rigorous evaluation of EGCG’s pharmacokinetics, tissue-specific bioavailability, and biotoxicity before clinical translation.

## Data Availability

The datasets presented in this study can be found in online repositories. The names of the repository/repositories and accession number(s) can be found in the article/supplementary material.
